# Heterologous expression of the gene for chlorite dismutase from *Ideonella dechloratans* is induced by an FNR‐type transcription factor

**DOI:** 10.1002/mbo3.1049

**Published:** 2020-04-22

**Authors:** Maria Rova, Miriam Hellberg Lindqvist, Thijs Goetelen, Shady Blomqvist, Thomas Nilsson

**Affiliations:** ^1^ Department of Engineering and Chemical Sciences Karlstad University Karlstad Sweden

**Keywords:** anaerobic induction, chlorate reduction, chlorite dismutase, FNR, horizontal gene transfer

## Abstract

Regulation of the expression of the gene for chlorite dismutase (*cld*), located on the chlorate reduction composite transposon of the chlorate reducer *Ideonella dechloratans*, was studied. A 200 bp upstream sequence of the *cld* gene, and mutated and truncated versions thereof, was used in a reporter system in *Escherichia coli*. It was found that a sequence within this upstream region, which is nearly identical to the canonical FNR‐binding sequence of *E*. *coli*, is necessary for anaerobic induction of the reporter gene. Anaerobic induction was regained in an FNR‐deficient strain of *E. coli* when supplemented either with the *fnr* gene from *E. coli* or with a candidate *fnr* gene cloned from *I. dechloratans*. In vivo transcription of the suggested *fnr* gene of *I. dechloratans* was demonstrated by qRT‐PCR. Based on these results, the *cld* promoter of *I. dechloratans* is suggested to be a class II‐activated promoter regulated by an FNR‐type protein of *I. dechloratans*. No *fnr*‐type genes have been found on the chlorate reduction composite transposon of *I*. *dechloratans*, making anaerobic upregulation of the *cld* gene after a gene transfer event dependent on the presence of an *fnr*‐type gene in the recipient.

## Graphical abstract

Activation of the chlorite dismutase (cld) gene, located in the chlorate composite transposon of the chlorate reducer *Ideonella dechloratans*, was studied in a transcriptional reporter system in an *fnr*‐deficient *Escherichia coli* strain. The results demonstrate that an *fnr*‐type regulator of *I. dechloratans*, not present in the chlorate composite transposon, induces this gene under anaerobic conditions. Implications for horizontal gene transfer of the capability of dissimilatory chlorate reduction are discussed.

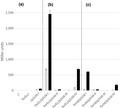

## 1 INTRODUCTION

Dissimilatory perchlorate‐ and chlorate‐reducing prokaryotes, collectively known as (per)chlorate‐reducing bacteria (PCRB), utilize reduction of perchlorate (ClO_4_^−^) and/or chlorate (ClO_3_^−^) in respiratory electron transport. Most PCRB are facultative anaerobes capable of using oxygen as terminal electron acceptor under aerobic conditions and (per)chlorate under anaerobic conditions. Reduction of the chlorine oxyanions occurs in the periplasm of the cell and is catalyzed by molybdoenzymes belonging to class II of the dimethyl sulfoxide (DMSO) reductase family (Magalon, Fedor, Walburger, & Weiner, 2011). Perchlorate reducers contain perchlorate reductase (PcrAB) which catalyzes both the reduction of ClO_4_^−^ to ClO_3_^−^ and the reduction of ClO_3_^−^ to chlorite (ClO_2_^−^) (Bender et al., 2005). Chlorate reducers have a chlorate reductase (ClrABC) instead of PcrAB and are only able to perform the reduction of ClO_3_^−^ to ClO_2_^−^ (Thorell, Stenklo, Karlsson, & Nilsson, 2003). All PCRB examined so far except the archaeon *Archaeoglobus fulgidus*, which uses sulfide as a chlorite scavenger (Liebensteiner, Pinkse, Schaap, Stams, & Lomans, 2013), have a chlorite dismutase (Cld). Clds, first characterized in the perchlorate‐reducing bacterium *Azospira oryzae* GR‐1 (van Ginkel, Rikken, Kroon, & Kengen, 1996), are heme *b*‐containing enzymes. They are highly efficient in decomposing chlorite to chloride and molecular oxygen (ClO_2_^−^→Cl^−^ + O_2_) and therefore essential in PCRB for detoxification. Also, the oxygen produced serves as an additional respiratory electron acceptor, increasing the utility of (per)chlorate as respiratory substrate.

(Per)chlorate‐reducing bacteria are thought to play important roles in the biogeochemical cycle of chlorine on Earth (Atashgahi et al., 2018). Most of the perchlorate and chlorate found in the environment today have an anthropogenic origin. Chlorate appears in wastewaters from the pulp and paper industry, and perchlorate is used in several applications, for example, in the manufacture of munitions. Contamination of soils, food, and freshwater reservoirs has become a threat to public health in, for example, the USA, India, and China (Kumarathilaka, Oze, Indraratne, & Vithanage, 2016), and bioremediation by the use of PCRB seems to be one of the best ways to decrease (per)chlorate load in the environment (Hatzinger, 2005; Ma, Bonnie, Yu, Che, & Wang, 2016). Other interesting biotechnological applications have also been suggested for PCRB based on their ability to produce molecular oxygen in anaerobic environments (Wang & Coates, 2017).

(Per)chlorate‐reducing bacteria are phylogenetically diverse, found mainly in Proteobacteria (Coates & Achenbach, 2004) but also in Firmicutes (Balk, Gelder, Weelink, & Stams, 2008; Balk et al., 2010) and Archaea (Liebensteiner et al., 2013). Several lines of evidence suggest that dissimilatory (per)chlorate reduction has been spread by horizontal gene transfer. PCRB are distributed over different classes, phyla, and even domains whereas their closest relatives typically are non‐PCRB. The phylogenetic tree of Cld does not overlap with that of 16S rDNA of PCRB (Bender, Rice, Fugate, Coates, & Achenbach, 2004; Maixner et al., 2008). In 13 perchlorate reducers examined, the Pcr operon (*pcrABCD*) and the gene for Cld (*cld*) were found on perchlorate reduction genomic islands (PRIs). Localization of PRIs in tRNA genes, presence of mobility genes close to the PRI core and inverted repeats at possible integration sites indicate integration of the PRI into the host genome (Melnyk & Coates, 2015; Melnyk et al., 2011). In a study of six chlorate reducers, the Clr operon (*clrABDC*) and the *cld* gene were found to be flanked by insertion sequences, which in five of the six strains were identified as sequences known to form composite transposons in other systems (Clark, Melnyk, Engelbrektson, & Coates, 2013).

Respiration of (per)chlorate is dependent on several proteins besides (per)chlorate reductase and chlorite dismutase. The integration of this metabolism into a new host requires that the necessary proteins either are carried on the transposable element or preexist in the recipient. Interestingly, the presence of accessory genes seems to differ between the PRIs of the perchlorate reducers and the chlorate reduction composite transposons of the chlorate reducers. While the PRIs studied by Melnyk (Melnyk & Coates, 2015) contained several accessory genes, some of which have been proven necessary for perchlorate reduction (Melnyk, Clark, Liao, & Coates, 2014), the chlorate reduction composite transposons examined by Clark (Clark et al., 2013) contained just a few genes in addition to chlorate reductase and chlorite dismutase. Most of the accessory genes identified on PRI cores belong to one of the four functional groups: transcriptional regulation, electron transport, oxidative stress resistance, or molybdenum cofactor biogenesis (Melnyk & Coates, 2015). The nature of the accessory genes reflects the functions needed in the host to be able to exhibit perchlorate reduction capacity, and the same functions should be required for chlorate reduction. Expression of the key enzymes is expected to be regulated by, for example, the availability of different electron acceptors. Suitable redox components must be present in the cell to deliver electrons from the membrane to the periplasmic reductase. Hypochlorite is produced as a byproduct of Cld activity (Hofbauer et al., 2014), and it is likely that the need for protection against oxidative stress increases during (per)chlorate reduction. A system for biogenesis and integration of the molybdenum cofactors of Pcr or Clr has to be present in the cell. Examination of required accessory genes and their genomic localization will give insights into the evolution of the transposable elements of these complex metabolic traits and facilitate the understanding of the requirements of a non‐PCRB recipient.

In this study, we have addressed the regulation of the expression of the *cld* gene in the chlorate reduction composite transposon of the chlorate reducer *Ideonella dechloratans*. We have previously shown increased mRNA levels and enzymatic activities of Cld when *I. dechloratans* is grown under anaerobic, chlorate‐reducing conditions compared to growth under aerobic conditions in the presence of chlorate, indicating that oxygen level or redox state is sensed by a regulatory factor (Hellberg Lindqvist, Johansson, Nilsson, & Rova, 2012). The only regulatory gene found in the composite transposon of *I. dechloratans* is a member of the ArsR family (Clark et al., 2013). However, most members of this family are involved in metal sensing making this regulator a less likely candidate for oxygen‐dependent regulation of *cld*. The results of the present study suggest a role for an FNR‐type regulator, not included in the chlorate reduction composite transposon, in activating the *cld* gene of *I. dechloratans* under anaerobic growth conditions. This is, to our knowledge, the first report of how a gene on a chlorate reduction composite transposon is regulated and also the first report of a functional FNR transcription factor in *I. dechloratans*.

## 2 MATERIALS AND METHODS

### 2.1 Strains, plasmids, and growth conditions

Bacterial strains listed in Table A1 were used as follows: *Escherichia coli* XL‐1 Blue and JM109 for cloning; *E. coli* RM101 (Sawers & Suppmann, 1992) as an *fnr*‐negative background for expression studies; *Ideonella dechloratans* (culture collection of Göteborg University, Göteborg, Sweden, CCUG 30977; Malmqvist et al., 1994) as a source of the *cld* promoter region (AJ296077.1) and an *fnr*‐type gene and its mRNA (img: 2510552075) and *E. coli* MG1655 (DSM 18039) as a source of an *fnr* gene (GeneID: 945908). The broad‐host‐range promoterless reporter vector pBBR1MCS‐2‐lacZ (Kan^R^; Table A2) was fused with different parts of the upstream region of the *cld* gene of *I. dechloratans* and used in RM101. pBR322 (Table A2) was used for cloning and expression of the *fnr* genes in RM101.

All liquid cultures were grown in shake incubator at 37°C and 200 rpm. Antibiotics were used when appropriate to a final concentration of 100 µg/ml ampicillin and/or 50 µg/ml kanamycin. For β‐galactosidase assay, *E. coli* RM101 was grown in a medium described in Constantinidou et al. (2006) containing minimal salts (Pope & Cool, 1982) supplemented with 0.4% glycerol, 40 mM sodium fumarate, 20 mM trimethylamine N‐oxide (TMAO), and 10% (v/v) Luria–Bertani. Cells were harvested in exponential growth at OD_600_ of 0.4–0.7 under both aerobic and anaerobic growth conditions. Aerobic cultures were prepared by picking single colonies of RM101 from fresh Luria–Bertani agar plates to overnight cultures in 3 ml of the medium described above and after 15–16 hr of growth 10 µl was used to inoculate 10 ml fresh medium in 100 ml Erlenmeyer flasks and grown for about 4 hr. For the anaerobic cultures, colonies were picked to starter cultures of 3 ml of the medium described above and grown for 2 hr. From these cultures, an inoculum was diluted about 4 × 10^6^ times in fresh medium to a final volume of 35 ml and grown overnight for 15–16 hr in completely filled, rubber‐sealed flasks. *I. dechloratans* was grown aerobically and anaerobically as described in Lindqvist, Nilsson, Sundin, and Rova (2015). *E. coli* MG1655, XL‐1 Blue, and JM109 were grown in Luria–Bertani medium.

### 2.2 Promoter constructs

**FIGURE 1:**
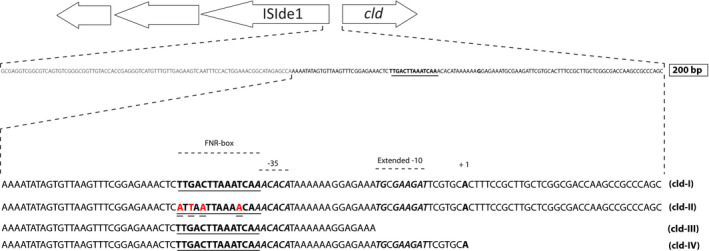
Part of the chlorate composite transposon of *Ideonella dechloratans* showing the sequence of the region from 31 to 230 bp upstream of the start codon of *cld* (GenBank AJ296077.1). The suggested binding sites for FNR and RNAP and the suggested transcription start of *cld* are indicated. The differences between the fragments used in this study are shown as the four double‐underlined bases that was changed in cld‐II and the exact 3′‐end of all four sequences (cld‐I‐IV)

A series of plasmids, p2cld‐I‐IV (Table A2; Figure 1), was created by insertion of different parts of the upstream region of the *cld* gene (AJ296077.1) from *I. dechloratans* into the reporter vector pBBR1MCS‐2‐*lacZ* (Fried, Lassak, & Jung, 2012). Genomic DNA from *I. dechloratans* was amplified by PCR primers listed in Table A3, the PCR products ligated with the reporter vector and the resulting plasmids transformed into *E. coli* XL‐1 Blue Supercompetent Cells (Agilent Technologies) by standard procedures. The following constructs were produced: p2cld‐I with a 200 bp upstream region of *cld*; p2cld‐III (151 bps from the same 5′‐end as the 200 bp segment to the last bp before a predicted −10 region of RNA polymerase binding) and p2cld‐IV (167 bp from the same 5′‐end as the 200 bp segment to +1 of the predicted transcription start). Mutagenesis of the 200 bp sequence was performed with QuickChange Lightning Site‐Directed Mutagenesis Kit (Agilent Technologies) according to the instructions from the manufacturer and with primers carrying four point mutations in the putative FNR box (Table A3), resulting in p2cld‐II. All constructs were verified by sequencing (Eurofins Genomics). Promoter constructs of pBBR1MCS‐2‐*lacZ* (p2cld‐I‐IV; Kan^R^) and the backbone plasmid itself were transferred from XL‐1 Blue to RM101 cells by electroporation. Each of the resulting RM101 clones went through another transformation in which pBR322 (Amp^R^) containing *fnr* from *E. coli*, *fnr* from *I. dechloratans* or without insert was transferred. This resulted in three different double transformants of each promoter construct. These double transformants were grown in the presence of both ampicillin (100 µg/ml) and kanamycin (50 µg/ml) to keep both plasmids.

### 2.3 Cloning of *fnr* genes from *E. coli* and *I. dechloratans*

The *fnr* gene from *E. coli* (GeneID: 945908) was cloned from strain K‐12, substrain MG1655. A sequence starting 226 bp upstream of transcription start and ending 251 bp downstream of stop codon was amplified with PCR primers shown in Table A3, ligated with pBR322 and transformed into *E. coli* XL‐1 Blue cells. The predicted protein sequence from this gene (UniProtKB:P0A9E5) was used as the query in a BLASTp search for a homologous sequence in the genome of *I. dechloratans* (img: 2510461017). A fragment including the best hit (img: 2510552075), starting 221 bp upstream of the proposed start codon and ending 77 bp after the stop codon, was amplified from genomic DNA with PCR primers shown in Table A3. PCR products were ligated into pBR322 and transformed into *E. coli* JM109 Competent cells (Agilent Technologies). The authenticity of the cloned sequences was verified by DNA sequencing (Eurofins Genomics). The *fnr*‐containing plasmids and pBR322 without insert were isolated and each transferred by electroporation into *E. coli* RM101 clones containing the different *cld* promoter constructs or a promoterless pBBR1MCS‐2‐*lacZ* as described in Section 2.2.

### 2.4 Quantitative real‐time PCR

The relative amount of mRNA from the *fnr*‐type gene of *I. dechloratans* (img: 2510552075) was estimated by qRT‐PCR. *I. dechloratans* was grown under aerobic and anaerobic conditions as in Lindqvist et al. (2015). Isolation of RNA and performance of qRT‐PCR was as described in Hellberg Lindqvist et al. (2012) using the gene‐specific primers listed in Table A3 and with each sample analyzed in duplicate. The specificity of primers could be confirmed by the observation of single bands after agarose gel electrophoresis of PCR products. The amount of mRNA from the target gene *fnr* was normalized to the reference gene 16S rRNA and presented as $\Delta C_{T}=\left(C_{\text{T target}}-C_{\text{T reference}}\right)$ . In addition to nontemplate controls, samples without reverse transcriptase were used as negative controls to verify successful genomic DNA removal.

### 2.5 β‐Galactosidase assays

RM101 cells were grown and harvested as described in Section 2.1. β‐galactosidase assays were performed according to Miller (1972) with centrifugation of the samples at 10,000 *g* for 3 min before measuring OD_420_ instead of recording OD_500_. For the assay, 25–500 µl of each cell culture was used. The β‐galactosidase activity in Miller units (MU) was calculated with the following formula:

$$\text{Miller units}=1,000\times \frac{{\text{OD}}_{420}}{t\times v\times {\text{OD}}_{600}}$$

The OD_600_ denotes the cell density before the assay, OD_420_ the absorbance of the *o*‐nitrophenol, *t* is the reaction time in minutes, and *v* is the culture volume in milliliters.

## 3 RESULTS AND DISCUSSION

### 3.1 FNR‐dependent expression from the *cld* promoter

We have previously shown that the expression of the *cld* gene of *I. dechloratans* increases 5–10 times in a chlorate independent manner when cultures are transferred from aerobic to anaerobic conditions (Hellberg Lindqvist et al., 2012). This suggests regulation by an oxygen‐ or redox‐sensing regulator. We have identified a 14 bp sequence centered 105.5 bp upstream of the start codon of *cld* that is identical in 9 out of 10 nucleotides with the canonical FNR box of *E. coli* (TTGACTTAAATCAA vs. TTGATNNNNATCAA), and which may serve as a regulatory sequence for an FNR‐type transcriptional regulator.

To explore a possible role for this sequence and FNR as a regulator, a 200 bp fragment spanning from 31 to 230 bp upstream of the start codon of *cld* and a mutated version of the same sequence were cloned into the promoterless reporter plasmid pBBR1MCS‐2‐lacZ creating the plasmids p2cld‐I and p2cld‐II, respectively (Figure 1). For p2cld‐II, the putative FNR‐binding sequence had been changed from TTGACTTAAATCAA to ATTAATTAAAACAA by introducing four point mutations (underlined). Mutations in these positions have been shown to severely inhibit the function of the FNR box in *E. coli* (Bekker et al., 2010). These reporter constructs were used in complementation studies in the Δ*fnr* strain *E. coli* RM101. RM101 cells were double‐transformed with one of three pBBR1MCS‐2‐*lacZ* derived plasmid: pBBR1MCS‐2‐*lacZ* as a negative control; p2cld‐I containing the wild‐type 200 bp promoter sequence, or p2cld‐II containing the mutated sequence, in combination with one of two pBR322 derived plasmid: pBR322 as a negative control or pBR322(*fnr*_Ec_) containing the *fnr* gene of *E. coli* with its promoter and regulatory sequences. The resulting six double transformants were grown aerobically and anaerobically in a medium containing fumarate and TMAO that supports the growth of *fnr*‐deficient *E. coli* also under anaerobic conditions (Constantinidou et al., 2006).

**FIGURE 2:**
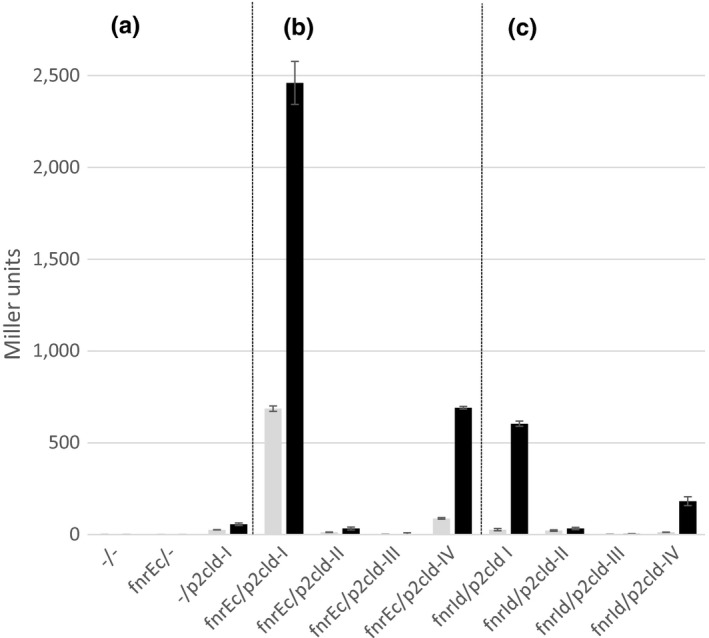
β‐galactosidase activity from *Escherichia coli* RM101 *fnr*‐deficient cells. Cells were doubly transformed with pBR322 with or without an insert of an *fnr* gene and the reporter plasmid pBBR1MCS‐2‐*lac Z* with or without the insert. The source of the *fnr* gene was either *E. coli* (fnrEc) or *Ideonella dechloratans* (fnrId). Inserts in pBBR1MCS‐2‐lac Z were a 200 bp insert from the upstream region of the *cld* gene from *I. dechloratans* (p2cld‐I); the same 200 bp upstream sequence but with 4 point mutations in the putative binding sequence of FNR (p2cld‐II); 151 bp from the 5′‐end of the 200 bp sequence, ending just before the predicted −10 region (p2cld‐III) and 167 bp from the 5′‐end of the 200 bp sequence, ending at +1 of the predicted transcription start (p2cld‐IV). Cells were grown at aerobic (gray) or anaerobic (black) conditions. (a) Controls lacking inserts (‐) in one or both backbone plasmids. Activities of controls not shown were as follows: fnrId/‐ no activity at either growth regime, ‐/p2cld‐II‐IV less activity compared to ‐/p2cld‐I. (b) Upstream regions of *cld* from *I. dechloratans* tested in cells complemented by *E. coli fnr*. (c) Upstream regions of *cld* from *I. dechloratans* tested in cells complemented by *I. dechloratans fnr*. Values are mean values from three independent measurements with three replicates in each. Error bars indicate standard error of mean (*SEM*)

Introduction of the 200 bp promoter region into Δ*fnr* RM101 cells in the absence of an *fnr* gene only slightly increased β‐galactosidase activity above background level under aerobic as well as anaerobic growth conditions (‐/p2cld‐I in Figure 2a). Cotransformation of the promoter sequence and the *fnr*_Ec_ gene, however, resulted in a pronounced increase in β‐galactosidase activity with four times higher expression under anaerobic compared to aerobic conditions (fnr_Ec_/p2cld‐I in Figure 2b). This indicates that the 200 bp fragment from *I. dechloratans* contains both a promoter region and FNR‐dependent regulatory sequence(s) and that those elements are functional in an *E. coli* background.

The mutated promoter of p2cld‐II showed low activity of about half the values of p2cld‐I under both growth conditions when tested alone and in contrast to what was found with p2cld‐I, the addition of the *fnr*_Ec_ gene did not have any effect on activity from the reporter gene (Figure 2b). This complete absence of an FNR‐dependent induction as a result of changing the four nucleotides strongly suggests that the FNR box‐like sequence from *I. dechloratans* binds a regulator.

FNR is expected to be mostly in its monomeric form under aerobic conditions, resulting in poor DNA‐binding capacity. FNR‐dependent expression of the reporter gene was however observed also under aerobic conditions. This may be a consequence of a high enough amount of activated dimers to activate the reporter gene as a result of multiple copies of the *fnr*_Ec_ gene (Mettert & Kiley, 2005).

### 3.2 A putative binding site of RNA polymerase in the *cld* promoter

The most common position of a single activating FNR‐binding sequence in *E. coli* appears to be at class II sites, which is centered around −41.5 (Myers et al., 2013). A previous attempt to identify the transcriptional start site (TSS) of *cld* resulted in a possible TSS at 86 bp upstream of the start codon (Thorell, Karlsson, Portelius, & Nilsson, 2002). However, this would place the FNR‐binding sequence in a position centered at −19.5 which is incompatible with the role of FNR as a transcription activator since it would place the regulator between the −35 and −10 binding regions of σ^70^ RNA polymerase (RNAP), preventing binding of the polymerase. We therefore hypothesized a TSS at 41.5 (±4) nt downstream of the center of the FNR‐binding site and searched for possible −10 and −35 binding regions. We found that with a TSS at exactly 41.5 bp downstream of the center of the binding site, the −12 to −7 sequence is GAAGAT with 3 out of 6 nt (underlined) identical to the *E. coli* consensus sequence TATAAT (Figure 1). Interestingly, two of these, −7 T and −11 A (bold), are identical to the two positions found to be of greatest relevance for σ^70^ RNAP binding in *E. coli* (Heyduk & Heyduk, 2014). Spaced by the optimal distance of 17 bp from the −10 hexamer and overlapping by 1 nt with the FNR site is the hexamer AACACA with 3 positions (underlined) corresponding to the consensus sequence TTGACA of *E. coli*. Promoters with weak −35 regions will often have TG in position −15 to −14, so‐called extended −10 promoters (Mitchell, 2003). TG is found at −15 to −14 in the analyzed sequence. Thus, the described sequence seems to fulfill the requirements of a σ^70^ RNAP‐binding site and the A at 41.5 nt downstream of the center of the FNR‐binding site could be a TSS.

To test this hypothesis, we made two truncated versions of the 200 bp promoter. The first version started from the same 5′‐end as the 200 bp sequence and ended at position −16 counted from the hypothesized TSS, that is, just before the T of the TG in the extended −10 region. The second version started from the same 5′‐end and ended at position +1, thus including the suggested binding site for RNAP and the TSS (Figure 1). The truncated promoter fragments were inserted in pBBR1MCS‐2‐*lacZ*, resulting in plasmids p2cld‐III (lacking the putative −10 region and downstream sequences) and p2cld‐IV (containing the putative −10 region and TSS). RM101 cells were doubly transformed with p2cld‐III or p2cld‐IV and pBR322 or pBR322(*fnr*_Ec_), and transcription of each construct was measured as β‐galactosidase activity. It was found that p2cld‐III could not support transcription in any of the tested conditions, that is, all combinations of aerobic or anaerobic growth with or without FNR (Figure 2). This shows that the functional promoter was lost in this construction. On the contrary, p2cld‐IV followed the same pattern as p2cld‐I although the absolute Miller values were lower (Figure 2). It can be concluded that the region hypothesized to contain a −10 sequence and a TSS is necessary for transcription. Based on homology with FNR‐ and RNAP‐binding sites of known class II promoters, it seems likely that this sequence binds RNAP also in *I. dechloratans*. The sequence downstream of the suggested TSS is not a requirement for transcription but seems to increase transcription since β‐galactosidase activity was lower for cells containing p2cld‐IV compared to p2cld‐I.

### 3.3 Cloning and characterization of an *fnr*‐type gene of *I. dechloratans*

**FIGURE A1:**
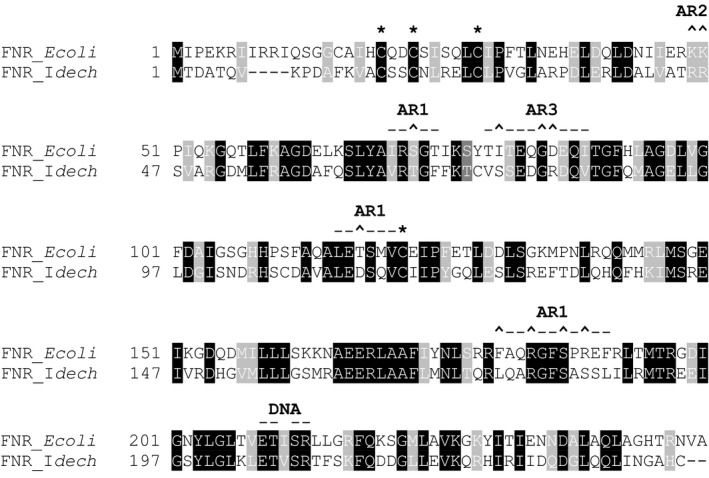
Alignment of the sequences of *Escherichia coli* FNR (UniProtKB:P0A9E5) and the homolog found in the genome of *Ideonella dechloratans* (img: 2510552075). Regions of the *E. coli* sequence that have been found to have special functions are indicated as: *, for the cysteine residues involved in the formation of the iron–sulfur cluster; “AR1‐3,” for activating regions 1–3 that interact with RNAP and “DNA” for residues involved in binding to the FNR recognition sequence. Residues shown to be important for activation of class II promoters are indicated by ^

The capability of FNR_Ec_ to recognize binding sequences from *I. dechloratans* and to regulate the expression in the reporter constructs raises the question of whether a corresponding protein is present in *I. dechloratans*. To investigate this, a BLASTp search of the genome of *I. dechloratans* Anox B ATCC 51718 available in the IMG database (https://img.jgi.doe.gov/cgi‐bin/m/main.cgi) was performed. The sequence of FNR from *E. coli* K12 (P0A9E5) available in the UniProtKB database (http://www.uniprot.org/) was used as the query. A potential homolog (E value: 10^−62^) corresponding to a 244 amino acid long sequence annotated as a cAMP‐binding protein (img: 2510552075) was found localized downstream of a gene cluster that encodes a high‐affinity *cbb*_3_‐type oxidase. This sequence, hereafter called FNR_Id_, showed 44% identity with FNR_Ec_ and contained four cysteine residues in positions identical to the iron–sulfur cluster binding cysteines in FNR_Ec_ (Figure A1). A sequence spanning from 221 bp upstream of the proposed start codon and ending 77 bp after the stop codon was amplified by PCR, cloned into pBR322, forming pBR322(*fnr*_Id_), and used to complement Δ*fnr* RM101 clones transformed with p2cld‐I‐IV. Transcription was measured as β‐galactosidase activity.

It was found that FNR_Id_ was able to induce the expression of the reporter gene from the 200 bp upstream region of *cld* under anaerobic conditions (Figure 2c). Further, *fnr*_Id_ did not support the expression of the reporter gene when cotransformed with p2cld‐II, which means that FNR_Id_ can be expected to bind to its recognition sequence similarly as FNR_Ec_. No activity could be detected under any growth condition when the −10 region of the promoter identified for activation by FNR_Ec_ was missing (p2cld‐III), whereas anaerobically grown cells containing the −10 region and the suggested TSS (p2cld‐IV) showed activity upon complementation by FNR_Id_ in agreement with the findings for FNR_Ec_. It can be concluded that FNR_Id_ can bind the regulatory sequence homologous to an FNR box in the *cld* promoter and that this leads to expression from the *cld* class II promoter identified above. This suggests a role for FNR_Id_ in activating the *cld* gene also in *I. dechloratans*.

To explore if FNR_Id_ can be of physiological significance, relative mRNA levels were estimated by quantitative real‐time PCR (qRT‐PCR) in RNA preparations from cells of *I. dechloratans* grown under aerobic or anaerobic conditions. 16S rRNA was used as a reference as in Hellberg Lindqvist et al. (2012). The *fnr*_Id_ gene was found to be transcribed to the same relative level irrespective of growth regime with a ∆C_T_ value of 15.5 under aerobic and 15.6 under anaerobic growth, supporting a physiological role for the protein. This expression pattern is comparable to *E. coli* in which roughly equal amounts of FNR protein is known to be present independent of oxygen level (Sutton, Mettert, Beinert, & Kiley, 2004; Unden & Duchene, 1987). *fnr*_Ec_ is negatively autoregulated due to an FNR‐binding site spanning the TSS (Spiro & Guest, 1990). It could be hypothesized that also *fnr*_Id_ is autoregulated since an analysis with Virtual Footprint (Munch et al., 2005) of the upstream region of the *fnr*_Id_ gene for binding sites of RNAP and FNR resulted in a possible promoter at 96–124 bp and an overlapping FNR‐binding site at 111–124 bp upstream of the proposed start codon.

The level of expression of the reporter gene resulting from the action of FNR_Id_ was only about 25% of that seen with FNR_Ec_ but the difference between aerobic and anaerobic expression was more pronounced since the addition of pBR322(*fnr*_Id_) did not affect aerobic expression (Figure 2). This indicates that FNR_Id_ is not as efficient as FNR_Ec_ in activating transcription of the reporter gene in the *E. coli* background. There can be several reasons for this. Expression of the two FNR proteins is dependent on their endogenous promoters and the *fnr*_Ec_ promoter may be more efficient in the *E. coli* host resulting in a higher concentration of FNR_Ec_ compared to FNR_Id_. Interactions with the RNA polymerase from *E. coli* may be weaker for FNR_Id_ compared to FNR_Ec_, resulting in a lower overall expression rate of the reporter gene.

To characterize FNR_Id_ further, we aligned the amino acid sequence with that from FNR_Ec_ and analyzed it for known FNR specific motifs. In Figure A1, it can be seen that FNR_Id_ has four cysteines in positions identical to the four cysteines that bind the [4Fe‐4S]^2+^ cluster necessary for dimerization and sensing the oxygen level in FNR_Ec_. Further, the sequence ETxSR that binds the TTGAT recognition sequence (Bell, Gaston, Cole, & Busby, 1989; Matsui, Tomita, & Kanai, 2013) is found in a position in FNR_Id_ exactly corresponding to that in FNR_Ec_, consistent with the capacity of FNR_Id_ to recognize and bind the FNR recognition sequence preceding the *cld* gene. FNR_Id_ also shows partial homology to FNR_Ec_ in the three activating regions (ARs) of FNR proteins that have been shown to mediate protein–protein contacts between FNR and RNAP (Blake, Barnard, Busby, & Green, 2002; Lamberg, Luther, Weber, & Kiley, 2002; Weber, Vincent, & Kiley, 2005). Some of the amino acids identified as crucial for the contact between FNR and the RNAP for activation at a class II promoter differ between FNR_Id_ and FNR_Ec_ (Figure A1). This can be expected to lead to the lower affinity of RNAP_Ec_ for FNR_Id_ compared to FNR_Ec_ and may, at least in part, explain the relatively low activity of FNR_Id_ observed in *E. coli* RM101 cells.

An oxygen‐sensing FNR‐type protein may be the only activator needed in vivo for the observed anaerobic induction of *cld* but it is also possible that, in accordance with many other FNR‐activated genes, there are additional regulators affecting transcription of *cld* in *I. dechloratans*. Previously, we have shown that chlorate does not induce expression of *cld* in vivo in *I. dechloratans* under aerobic conditions but it cannot be ruled out that chlorite or chlorate acts as a signal molecule together with a second regulator under anaerobic conditions.

### 3.4 Implications for horizontal gene transfer of chlorate respiration

The capacity for chlorate respiration is widely distributed in the Proteobacteria phylum. Sequence analyses of chlorate‐reducing bacteria from different classes of Proteobacteria have shown the presence of insertion sequences enclosing the region containing the *cld* gene and the *clrABDC* operon, indicating the possibility of horizontal gene transfer through transposable elements (Clark et al., 2013). However, the complex process of chlorate respiration also requires specific biogenesis and electron delivery pathways as well as protection and regulatory systems, depending on several genes not included in the proposed transposable element. The chlorate composite transposon of *I*. *dechloratans* contains only three genes in addition to the *clr* operon and the *cld* gene that may be of relevance for chlorate respiration. Those are *cyc*, a c‐type cytochrome (Bohlin, Bäcklund, Gustavsson, Wahlberg, & Nilsson, 2010; Lindqvist et al., 2015), *mobB*, that may have a role in molybdopterin cofactor synthesis (Bohlin et al., 2010), and *arsR*, a putative transcriptional regulator (Clark et al., 2013). The *cyc* gene has been cloned and its gene product characterized and tested as electron donor to Clr in vitro (Bohlin et al., 2010). However, a function for it in chlorate respiration could not be established. Instead, cyt *c*‐Id1, a c‐type cytochrome not included in the chlorate composite transposon, was shown to be able to donate electrons to Clr in vitro (Bäcklund & Nilsson, 2011).

The present study suggests that the *cld* gene of *I*. *dechloratans* is induced by an FNR‐type transcription factor, FNR_Id_, not studied before*.* The chlorate composite transposon of *I*. *dechloratans* does not contain an *fnr* gene, and participation of the *cyc* gene in chlorate reduction is questionable. The lack of accessory genes on the chlorate composite transposon of *I. dechloratans* makes the success of a transfer event of this sequence dependent on the existing genetic makeup of the recipient and may limit the range of possible recipients. Oxygen‐sensing FNR‐type transcriptional factors are however widespread, and the promoter structure of the *cld* gene appears to belong to the type II class of FNR‐dependent promoters, implying that regulation of the anaerobic induction of the *cld* gene of *I. dechloratans* can be performed in a wide range of host strains. It can be noticed that another chlorate‐reducing β‐Proteobacterium, *Alicycliphilus denitrificans*, contains a chlorate composite transposon nearly identical to that in *I. dechloratans* (Clark et al., 2013). This shows that at least one relatively recent and successful transfer event of chlorate‐reducing capability has been enabled by a chlorate composite transposon similar to that in *I. dechloratans*.

Further studies to reveal the complete set of genes needed for chlorate respiration will give a deeper understanding of the physiological prerequisites for and evolution of chlorate and perchlorate respiration.

## CONFLICT OF INTEREST

None declared.

## AUTHOR CONTRIBUTION

**Maria Rova:** Conceptualization (equal); investigation (equal); project administration (equal); writing – original draft (lead); writing – review and editing (equal). **Miriam Hellberg Lindqvist:** Investigation (equal); writing – review and editing (equal). **Thijs Goetelen:** Investigation (equal); writing – review and editing (equal). **Shady Blomqvist:** Investigation (equal); writing – review and editing (equal). **Thomas Nilsson:** Conceptualization (equal); project administration (equal); writing – review and editing (equal).

## ETHICS STATEMENT

None required.

## Data Availability

All data generated or analyzed during this study are included in this published article.

## APPENDIX 1

**Table A1:** Bacteria strains used in this study

Bacterial strains	Genotype or relevant features	Reference or source
*Escherichia coli* XL‐1 Blue	*lac* [F′ *proAB lacIqZΔM15* Tn*10* (Tet^r^)]	Agilent Technologies
*E. coli* RM101	MC4100 *Δfnr Δ(argF‐lac)U169*	Sawers and Suppmann (1992)^a^
*Ideonella dechloratans*		Malmqvist et al. (1994)
*E. coli* MG1655	K‐12	DSM 18039
*E. coli* JM109		Agilent Technologies

^a^ *E. coli* strain RM101 was kindly donated by Prof. K. J. Hellingwerf.

**Table A2:** Plasmids used in this study

Plasmid	Genotype or relevant features	Reference or source
pBBR1MCS‐2‐*lacZ*	Broad‐host‐range reporter vector, ^a^ND, promoterless *lacZ* gene(Kan^R^)	Fried et al. (2012)^b^
p2cld‐I	pBBR1MCS‐2‐*lacZ* with 200 bp insert from 31 to 230 bp upstream start codon of *cld*	This work
p2cld‐II	Same as p4cld‐I except for 4 point mutations in predicted FNR box	This work
p2cld‐III	pBBR1MCS‐2‐*lacZ* with 151 bp insert from 80 to 230 bp upstream start codon of *cld*	This work
p2cld‐IV	pBBR1MCS‐2‐*lacZ* with 167 bp insert from 64 to 230 bp upstream start codon of *cld*	This work
pBR322	Cloning vector, ColE1‐based replicon (Tet^R^)(Amp^R^)	Thermo Fischer Scientific
pBR322(*fnr*_Ec_)	pBR322 with *fnr* gene (GeneID: 945908) and upstream sequences from *Escherichia coli* MG1655	This work
pBR322(*fnr*_Id_)	pBR322 with *fnr* gene (img: 2510461017) and upstream sequences from *Ideonella dechloratans*	This work

Abbreviations: Amp^R^, ampicillin‐resistant; Kan^R^, kanamycin‐resistant; Tet^R^, tetracycline‐resistant.

^a^ ND, the incompatibility group of pBBR1MCS vectors has not been defined; compatible with IncP, IncQ, and IncW group vectors, and with ColE1 and P15a based replicons.

^b^ pBBR1MCS‐2‐*lacZ* was kindly donated by Prof. K. Jung.

**Table A3:** Primers used in this study. Restriction enzyme sites added to the primers are underlined

	Forward primer (5′–3′)		Reverse primer (5′–3′)	Product size (bp)
Amplification of upstream region of *cld* of *Ideonella dechloratans*				
cld‐I	GGGCGAATTCGCGAGGTCGGCGTCAGT		GCGCAAGCTTGCTGGGCGGCTTGGTC	220
cld‐III	GGGCGAATTCGCGAGGTCGGCGTCAGT		GGCGCGTCGACTTTCTCCTTTTTTATGTGTTTGATT	172
cld‐IV	GGGCGAATTCGCGAGGTCGGCGTCAGT		GCGCGTCGACTGCACGAATCTTCGCATTTCTCCT	187
Mutagenesis of the putative FNR box with plasmid p4cld‐I as template				
cld‐II	CCAAAAATATAGTGTTAAGTTTCGGAGAAACTCATTAATTAAAACAAACACATAAAAAAGGAGAAATGCGAAGATTC		GAATCTTCGCATTTCTCCTTTTTTATGTGTTTGTTTTAATTAATGAGTTTCTCCGAAACTTAACACTATATTTTTGG	
Amplification of *fnr* genes from *Escherichia coli* or *I. dechloratans*				
*fnr E.c.*	GGCAAGCTTAGGCGGAGTTCAGCGAAAAG		CGCGGATCCTGCCACCAATCCGTTGATGT	1,279
*fnr I.d.*	GGGCGAATTCCGTCGAATTGCCGCAACAAG		GCGCGGATCCCTCACAAGACCCCAGGGATG	1,053
qRT‐PCR				
16SrRNA		CATCGGAACGTGCCCAGTAGTG	TGACATCGGCCGCTCCAATAG	119
*fnr*		CGCCTTCCTGATGAACCTGA	TAGCTGCCGATTTCTTCCCG	96
